# Ethnobotanical inventory of medicinal plants used by Cashinahua (*Huni Kuin*) herbalists in Purus Province, Peruvian Amazon

**DOI:** 10.1186/s13002-023-00586-4

**Published:** 2023-05-12

**Authors:** Jana Horackova, Maria Elena Chuspe Zans, Ladislav Kokoska, Naji Sulaiman, Zoyla Mirella Clavo Peralta, Ludvik Bortl, Zbynek Polesny

**Affiliations:** 1 Department of Crop Sciences and Agroforestry, Faculty of Tropical AgriSciences, Czech University of Life Sciences Prague, Kamýcká 129, Praha – Suchdol, 165 00 Czech Republic; 2 Departamento Académico de Ingeniería Civil y Ciencias Básicas, Universidad Nacional Intercutural de Quillabamba, El Arenal s/n, Cusco, Peru; 3 Instituto Veterinario de Investigaciones Tropicales y de Altura, Universidad Nacional Mayor de San Marcos, Jr. Daniel Alcides Carrión 319, Pucallpa, Peru; 4 Prague Botanical Garden, Trojská 800, 171 00 Prague, Czech Republic

**Keywords:** Ethnobotany, Ethnomedicine, Indigenous people, Traditional knowledge, Peru

## Abstract

This study aims to document the diversity of medicinal plants used by the Cashinahua people (also known as *Huni Kuin*) of the Curanja River, as well as describe and compare their uses with pharmacological and phytochemical records from previously published studies. The ethnic has been studied to a limited extent from an ethnobotanical perspective. The study area is located in the Ucayali region, eastern Central Amazon, where ancestral knowledge is preserved due to the limited accessibility of the region. Between November 2010 and June 2015, a total of 11 months were spent on the survey, which included a short-term visit to complete voucher specimen collection and taxonomic identification. We conducted semi-structured interviews with 10 Cashinahua traditional healers and 10 midwives. Vernacular names, ethnomedicinal uses, plant parts used and forms of preparation and administration were recorded. Ethnopharmacological, pharmacological and phytochemical uses were checked through survey of the previously published papers indexed on Web of Science databases between 2018 and 2022. We obtained data on 467 plant taxa, among which we highlighted 79 species unreported or rarely cited for medicinal use or phytochemical analysis. These species were spread over 60 genera and 42 botanical families, with Acanthaceae being the most represented. Leaves were used the most frequently (93.56%). Among the 79 species, the most reported therapeutic activities involved pregnancy and birth disorders (13.84%), followed by poisonings, infections and infestations. The predominant application form was external (87%). Our study indicates that there are locally valuable species that have not yet been studied for their medical potential*.*

## Introduction

Peru is one of the ten most biologically and culturally diverse countries in the world and is home to the second largest rainforest on the planet. The Amazon covers most of the country (57.9%), although only a small proportion of the population lives in this area—some 2.2 million people, or 9% of the country’s population. Presently, there is information on 55 indigenous groups in Peru, 51 of which live in the Amazon [1]. Peruvian tropical rainforests contain 23% of known tropical plant taxa [2], which constitutes the largest collection of vascular flora in Peru, with a total of 19,147 species, of which 7,590 are endemic [3]. This diversity represents the primary source of food, medicine, energy, crafts, dyes, fibres, art, rituals and symbols for human groups in Amazonia [4]. Reports on the number of medicinal species in Peru vary from the nearly 3,000 species mentioned by Mostacero et al*.* [5] to the 1,100 species described by Agapito and Sung [6], while Antonio Brack Egg [7] reported an intermediate value of 1,400 species. With such huge biodiversity, it is surprising how small a percentage of these plants have thus far been scientifically studied. Graham [8] states that the lack of regionally consistent data is, at least in Amazonia, the rule, and this represents a challenge for ethnobotanists and ethnobiology, which must find a way to resolve this important lack of continuity.

One of the main tributaries of the Amazon, the Purus River, originates in south-eastern Peru, one of the most diverse and unexplored parts of the Amazon Forest, creating, together with the Madre de Dios region of Peru and Brazil, a huge corridor for life and culture. Nearly 80% of the territory of Purus Province is under protection, with Alto Purus National Park, at more than 2.5 million hectares, standing out as the largest protected area in the country. The Alto Purus River basin is an indigenous territory inhabited by peoples belonging to the Panoan and Arawakan linguistic families. Approximately 80% of the population in this area is indigenous, making this watershed a centre of cultural diversity, as at least eight ethnic groups, namely the Cashinahua, Chaninahua, Mastanahua, Sharanahua (Panoan groups which native auto denomination is *Huni Kuin*, with phonetic variation), Amahuaca (also Panoan group), Culina, Asháninka and Yine (Arawakan groups), make up the communities of the Alto Purus River basin [9]. In addition, there are several Territorial Reserves, located in the most inaccessible areas near the headwaters of rivers, protecting the last virgin regions of the planet where nomadic groups identified as in a state of “voluntary isolation” and initial contact hunt, fish and gather.

The Cashinahua lives near the headwaters of the Jurua and Purus river systems: the former in the state of Acre, north-western Brazil, and the last in Purus Province in south-eastern Peru. The Peruvian Cashinahua represent the culturally more conservative part of this ethnic group whose ancestors migrated to Peru around a century ago fleeing a conflict in a rubber plantation on the Envira River (Brazil), where they work in rubber plantations [10]. The group represents 0.4% of the indigenous inhabitants recorded in Peru [11], with an estimated population of 1,831 [12]. A large part of the ethnic group inhabiting the banks of rivers and streams on the Brazilian side of the border has been the subject of several ethnobotanical studies [13, 14].

Until the rubber industry reached this remote part of the rain forest, the Cashinahua remained a completely isolated self-sufficient group at the headwaters of the Curanja, Envira and Jurua rivers between the territories of Peru and Brazil [15]. This Indigenous people are locally associated with the forest and are known to be mostly dependent on forest resources both in the provision of food and medicine. Here, most plant specialists gather their plants in the nearby forest having emergency supply plants planted in forest gardens in the vicinity of the community or grown around forest trails. Most families living along the Curanja River, as in all rural communities of the province, depend on subsistence agriculture and more specifically on the hunting and non-timber forest product collection. Through their close contact with, and dependence on, local biodiversity, they have acquired a thorough understanding of the physical and chemical properties of plants in the surrounding forest and have developed the ability to classify and exploit this incredibly diverse resource [15]. However, those who still know and use plants to heal themselves and others are slowly disappearing and usually have no successors. Access to Western medicines and the arrival of Christian missionaries (Catholic and Protestant) since the 1950s [16] contributed to the rejection of the socio-cultural knowledge on the use of some traditionally utilized plants. The Cashinahua people had a subsistence livelihood and lived in voluntary isolation less than 70 years ago. In a previous study, Graham [15] reports that within a few generations the society of this indigenous group has transformed from being almost entirely self-sufficient and isolated to increasingly dependent on the outside world for highly prized and essential goods, including medicines. Medical pluralism and the apparent ambivalence among younger generations about the value of their ancestral traditions endanger the survival of indigenous knowledge on the use of medicinal plants.

Dengue fever, leishmaniasis, schistosomiasis, scabies and intestinal parasites are among the more than 20 poverty-related pathologies that occur in tropical regions and are categorized as neglected tropical diseases by the Pan American Health Organization. Similar to the majority of tropical rural environments, Purus Province also faces other health issues that are predominantly nutritional and infectious. Due to the areas’ remoteness and lack of conventional medical care, the majority of residents in these isolated areas rely on natural medicinal resources and, most of the time, treat these ailments with traditional indigenous medicinal plants.

Traditional indigenous knowledge systems and biological experiences accumulated over generations have made a great and important contribution to studies on Amazonian biodiversity. Different authors have contributed to the study of ethnobotany in Ucayali, including Tournon [17, 18, 19], who described the current use of medicinal plants by the Shipibo-Conibo, the Panoan ethnic group prevalent in the Ucayali region. The Shipibo healer Guillermo Arévalo Valera along with the Swedish ethnopharmacologist Anders Hansson have co-founded a project called *Aplicación de Medicina Tradicional* (AMETRA), which has sought to revive the traditional medicine practices of the Shipibo-Conibo people. Valera and Hansson [20] have published a book on medicinal plants and their beneficial properties to health. The book is considered the first publication written by a Shipibo healer that contains descriptions of a considerable number of medicinal plants along with their Shipibo names and forms of use; however, the book did not include complete taxonomic identifications of plant species. Polesna et al. [21] documented traditional ethnobotanical knowledge related to the uses of 30 plant species belonging to 18 families in the traditional medicine of the mestizo and Shipibo-Conibo people in the Ucayali region. Several of the species documented as widely used in the region coincide with the plants that are the subject of our study.

Anthropological investigations in the region of Purus began in 1960 and continue today, giving rise to the publication of a wide variety of ethnographic material, some of which contains detailed information on the subsistence activities and specialized ethnobotanical practices of natives [22, 23, 24, 25]. Literature on the Cashinahua ethnic group (also known as *Huni Kuin* since late 1990) has been produced mainly in the form of ethnographic studies, while ethnobotanical studies are rather scarce. Sporadic ethnobotanical information on plants used by these indigenous peoples can be found in catalogues of useful plants made by Catholic and Protestant missionaries [26, 27].

Peruvian Cashinahua knowledge of useful plants is profound but remains poorly investigated. Therefore, ethnobotanical studies are necessary to have at least a basic idea of the relative importance of the available knowledge [15].

The general aim of our study is to contribute to the understanding and preservation of part of the national cultural heritage by means to document and evaluate the traditional use of medicinal plant species, as well as to assess the homogeneity of traditional knowledge of the informants and determine culturally important species. We specifically aim to highlight a set of medicinal plants that have been studied only minimally, or not at all, from a perspective of their bioactive constituents. In order to understand the degree of similarity of the species used by other ethnic groups, another objective is a comparative analysis of the medicinal plant species used, based on similar studies from neighbouring regions.

## Background and methodology

### Study area

Ucayali is one of the five Amazonian regions of Peru. There are nearly a half million people residing in the department, with urban inhabitants accounting for 81.0% and rural inhabitants for 19.0% of the population. Ucayali is divided into four provinces with its administrative centre in Pucallpa. Purus is one of these provinces, which has 2,860 inhabitants and a population density of 0.16 inhabitants/km^2^ [12]. However, these estimates are inaccurate mainly due to the movement of indigenous people between Purus and neighbouring regions in Brazil. Because of its geographical position and the hydrographic profile of the area, the only direct connection between the province and the rest of the national territory is by air [28]. Purus is known for its vast biodiversity as a result of its location, which borders protected natural areas, such as Alto Purus National Park, Purus Communal Reserve, the Madre de Dios Territorial Reserve and the Mashco Piro Indigenous Reserve. The entire province remains without roads, a situation that minimizes the impact of the massive influx of mestizos that always causes habitat degradation [8, 15]. The Purus River and its tributaries are navigable by small boats, and these represent an essential means of communication.

According to the global map of terrestrial ecoregions [29], the province of Purus is included in the “Southwestern Amazon Moist Forests ecoregion”. About 90% of the study area was classified within the same soil unit, composed of Tropudalfs and Eutrochrepts [30]. The soils of terra firma localities contain an average of 52.5% sand, with a near neutral pH (6.3), and relatively few nutrients. A thin margin along the large rivers is classified as young alluvial soils, which are more fertile than those located at higher elevations and are considered to be of higher quality for agricultural use [9]. The climate of the area is hot and humid with an average annual precipitation range between 1800 and 2400 mm, with the main rainy season lasting from December to April. Temperatures do not fluctuate much over the year [31].

**Fig. 1:**
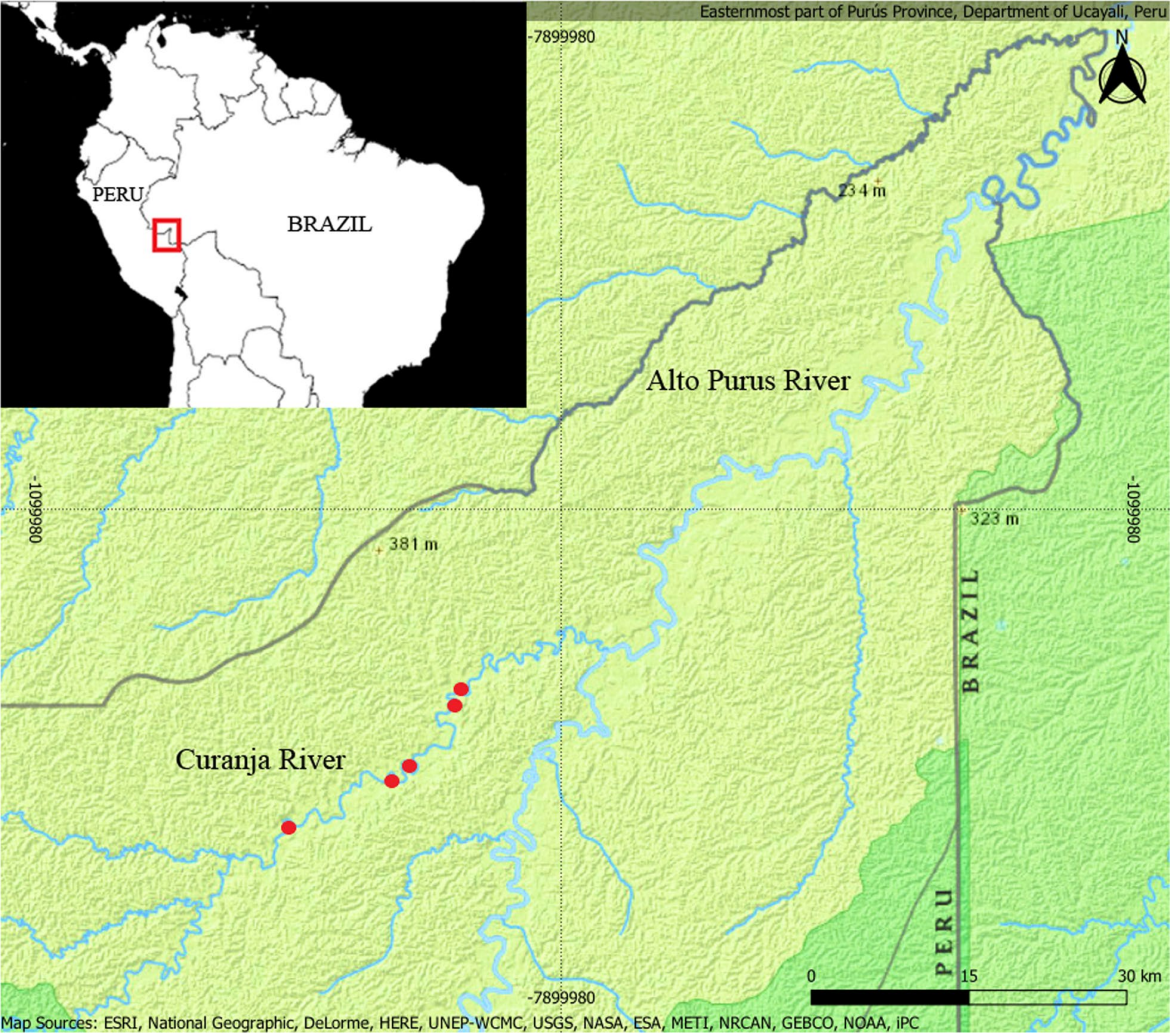
Map of study area. Red points indicate the data collection sites

In general, the Cashinahua people inhabit both banks of the Alto Purus River on both the Peruvian and Brazilian sides of border. In Peru, the Curanja River, a left affluent of the Alto Purus River, forms the buffer zone of the national park and is inhabited exclusively by the Cashinahua, which live in seven separate communities. The majority of research data presented in this study was collected in the surroundings of five communities plying the river Curanja (Fig. 1): Santa Rey with an approximate population of 96 inhabitants, Triunfo with 35, Colombiana with 65, Curanjillo with 31 and Nueva Vida with 31 inhabitants; with the base community being Colombiana. The communities are typically located on the high banks of the river, surrounded by primary rainforest.

### Ethnographic background

The Cashinahua people survive through a combination of subsistence activities such as hunting, fishing and tending small garden plots. Although the Cashinahua are primarily hunters, they rely on small-scale migratory slash-and-burn agriculture that coincides with the seasons of the rainforest. They practice four main crop systems: slash and burn, or swidden, agriculture that involves short-cycle crops, such as corn (*Zea mays* L.), cassava (*Manihot esculenta* Crantz), yam (*Dioscorea trifida* L.f.) and pumpkin (*Cucurbita maxima* Duchesne), which are cropped in regrown areas or virgin forests; beach agriculture, a distinctive activity among the Cashinahua community, with plantations of peanuts (*Arachis hypogaea* L.) and watermelons (*Citrullus lanatus* (Thunb.) Matsum. & Nakai); banana (*Musa* spp.) plantations by the river, where clay soils are predominant; and small gardens around their houses, mainly composed of aromatic and medicinal species. Traditionally, everything the Cashinahua used or consumed was harvested or produced locally and from locally available resources. The introduction of foreign goods and practices has produced only a slight modification in traditional subsistence patterns [16]. The basic economic and property-holding unit is the nuclear or polygynous family, and the basic social and political unit is the village. Each village is independent, with its own chief. Infrequent and irregular interactions between the villages, generally, are of a social or ceremonial nature.

Cashinahua plant specialists play a vital role in their community [15] and can identify a large number of plants and their medical uses. Kensinger [25, 32, 33] recorded the traditional medicine practices of the Cashinahua at the time of their contact in the 1950s and 1960s. Then Graham [15], as part of his dissertation thesis, described the uses of 109 medicinal plants in the Curanja River area. Ehringhaus [13] documented the medicinal use of 26 species of the Piperaceae family by Brazilian *Huni Kuĩ* (Kaxinawa) along the Jordão River. Comparison with the *Una Isi Kayawa* book [14] demonstrates that the cultural and medicinal practices of Peruvian and Brazilian parts of this ethnic group diverged more than a century ago as a result of their separate botanical and social environments. The interest in continuing to document the medicinal plants of this native group is due to the limited or lack of scientific reports regarding the medicinal use of these species, taking as a basis the small number of ethnopharmacological studies on the Peruvian Cashinahua.

### Ethnobotanical methods and assessment

The survey was conducted between November 2010 and June 2015 over a total of 11 months and a subsequent additional short-term visit to complete collection and taxonomic identification of voucher specimens. The participating communities were sampled on the basis of a series of communications with Cashinahua elders made during previous visits to the region (2007–2009), which resulted in a collaboration agreement with the Federation of Indigenous Communities of Purus (FECONAPU) and the appropriate authorizations from village chiefs and the research participants (prior informed consent), including oral consent for publication. The study site was composed of the last five Cashinahua communities along the upper reaches of the Curanja River bordering the uninhabited zone of Alto Purus National Park. The information was gathered via open and semi-structured interviews with 10 local informants widely recognized in Cashinahua communities as traditional healers and who proved to possess a high degree of plant knowledge [34]. Because of the limited information concerning plants used for gynaecological care by male study participants, an additional ethnobotanical survey with 10 female informants—midwives—was implemented, which focused on medicinal plant use in the process of family planning, gravidity, labour, the postpartum period, puerperium and new-born protection. The age of the informants ranged between 36 and 77 years. Of the 20 participants included in this study, 75% of them were between 60 and 80 years of age. Key informants provided botanical samples of medicinal plants, which were collected during forest walks, and semi-structured interviews were developed to discuss the uses of these plants, the plant parts used, and their preparation and application [35]. To collect plant material, field trips were taken with the practitioners to different vegetation zones in the vicinity of the study sites. Study participants were asked to mention and show on site any medicinal plants that they knew and used, providing information on the local name of the plant, important characteristics used to recognize the plant and dietary taboos. For each species, detailed photo documentation and ethnomedical information that included use, preparation, administration and healing concepts were recorded. Interviews were complemented with direct participant observation of the preparation methods and therapeutic practices associated with the collected species. Most information was noted and recorded in the *Hantxa Kuin* language and subsequently translated into Spanish with the help of a bilingual facilitator recruited from the community members. All voucher herbarium specimens were taxonomically identified and deposited in Peruvian institutions: complete sets of duplicates (Hor 1—590) were deposited in the herbaria of the Universidad Nacional Intercultural de la Amazonia (UNIA) and the Regional Herbarium of Ucayali (IVITA). The nomenclature used follows that of Plants of the World Online [36] and APG IV [37]. Quantification of ethnobotanical data was performed as indicated. First, the ethnobotanical information collected was converted into use reports (UR). One UR corresponds to an event where an informant mentions the use of a species to treat a particular disease category. In order to analyse the cultural importance of an individual species, major categories of uses based on the part of the human body affected by an illness (e.g. respiratory system, digestive system, muscular-skeletal system) were distinguished [38]. Systemic disorders (especially infections and inflammations) and culture-bound syndromes formed another category. The individual responses for each species in each of these categories were then summed, and the therapeutic uses of the plants were sorted and quantitatively assigned to these various categories. Most plant species were classified under several categories.

Informant consensus factor (ICF) was then calculated based on the quantification of ethnobotanical data. In traditional medical systems, the same plant species is often used to treat different unrelated ailments. To test the homogeneity of ethnomedical knowledge, the ICF proposed by Heinrich [39] was calculated to indicate whether or not there is agreement among respondents in the use of plant species in each disease category. The factor was calculated as: ICF = (Nur − Nt)/(Nur − 1), where Nur is the number of UR in each disease category and Nt is the number of species used in the same category by all interviewed informants. ICF values ranged from 0 to 1, where a high ICF value describes a high level of agreement among respondents, indicating that there is a well-defined criterion for selecting species used to treat a disease category. Following that, a medicinal plant overlap analysis was carried out. First, all suitable ethnobotanical studies from the Peruvian Amazon and neighbouring regions in Brazil and Bolivia [40, 41, 42, 43] were compared to the medicinal species in the study area. Following González-Tejero et al. [44], Jaccard’s similarity indices were calculated and the diversity of medicinal plants was compared. Jaccard’s index is calculated as [C/(A + B − C)] × 100, where A represents the number of species in sample A, B represents the number of species in sample B, and C represents the number of species that are shared by samples A and B.

This study allowed us to classify collected species taxonomically, and then contrast the existence of scientific information on their ethnopharmacological, pharmacological and phytochemical uses, selecting species without reports of use or surveys of active compounds within Web of Science databases between 2018 and 2022. On this basis, 79 species unreported or rarely cited for medicinal use were selected.

A compilation of all plant information in the *Hantxa Kuin* language was transcribed from recordings by a native speaker and returned to respondents in hard copy in 2018, along with another set containing photos and the local names of the collected plants, in the hope that it may prove useful in stimulating interest in traditional knowledge and cultural conservation within the local community. This ancestral knowledge, which was previously only accessible via oral tradition, is now becoming accessible to all of humanity.

## Results and discussion

We documented 467 plant taxa used for medicinal purposes by the Cashinahua community. In this paper, we present the general findings related to these 467 taxa with a main focus on 79 vascular plant species for which there is no documented medicinal use.

Out of the 467 taxa, most botanical samples were identified to species level, 105 to genus level, 6 to family level, while 12 taxa remained taxonomically unidentified. All the documented species are named in the *Hantxa Kuin* language. Some species were collected several times to complete fertile samples or were introduced by informants under a different vernacular name. We can observe, from a botanical perspective, an over-differentiation [45] of 72 plant species which bear two to four different local names. In six cases, under-differentiation was observed in which the identical vernacular name corresponded to different botanical species.

The 467 documented taxa belonged to 99 botanical families, the most predominant of which were Acanthaceae with 35 species, followed by Piperaceae (28 spp.), Rubiaceae (27 spp.), Araceae and Bignoniaceae (20 spp. each). The species most collected and used by informants were *Pseuderanthemum lanceolatum* (Ruiz & Pav.) Wassh. with 38 UR, *Leonia glycycarpa* Ruiz & Pav. and *Piper reticulatum* L. with 30 UR each, *Piper aduncum* L., *Uncaria tomentosa* D.C., *Tradescantia zanonia* (L.) Sw., *Abuta grandifolia* (Mart.) Sandwith, *Matisia cordata* Bonpl., *Pseuderanthemum congestum* (S.Moore) Wassh., *Drymonia tenuis* (Benth.) J.L.Clark, *Mascagnia eggersiana* (Nied.) W.R.Anderson and *Piper leucophaeum* Trel.

The most reported therapeutic activities were envenomation, particularly venomous snake and insect bites; infections and infestations, including herpes and conjunctivitis; the treatment of pregnancy and birth disorders, with the most prevalent uses being prenatal care, facilitating childbirth and accelerating labour; and digestive system disorders, for which the most frequently cited symptoms were diarrhoea, vomiting and constipation, and dental care. Almost all the taxa used in the treatment of culture-bound syndromes—unspecified socio-cultural use, family planning and religious use—also had normal therapeutic or prophylactic uses.

The most common forms of application were via external use, at 85.9%. Warm baths were the most prevalent (762 UR), which together with cold baths (89 UR) accounted for a total of 32.7% of the applications for external use. Direct application on the affected body part amounted to 34.4%, rubbing 15.1% and washing of the affected part 8.4%. The instillation of the juice of fresh leaves directly into the eye represented 5.6%. Oral administration accounted for 14.1% of the applications or 444 UR, which included the use of potions prepared by macerating pounded fresh plant material in water (64.6%, 287 UR), or decoctions (27%, 120 UR). Lastly, 1.1% of the recorded plant material, mostly leafy branches of *Couepia obovata* Ducke together with the leaves of the fern *Pityrogramma calomelanos* (L.) Link, burnt as a fumigant, was used to control infectious disease epidemics and to repel insects. The majority of our reported species (99%) were considered as wild, and they were usually collected in the nearby forest, along river banks and less often in anthropic environments (*chacras*) over quite a large gathering distance (5 km) from human settlements. In order to be ready for any emergency, each of the plant specialists maintained a small forest garden near the village. There, they transplanted some rare medicinal plants that are uncommon in the vicinity. Male plant specialists kept tree seedlings growing from natural reproduction around their paths well pruned so that they always had access to fresh leaves when needed. For the same reason, most samples collected from the trees were juveniles. Leaves were the most common plant part used (97%), explaining why the Cashinahua call their traditional medicine “*dau pei”*, leaf medicine.

### Predominant families and species

**Fig. 2:**
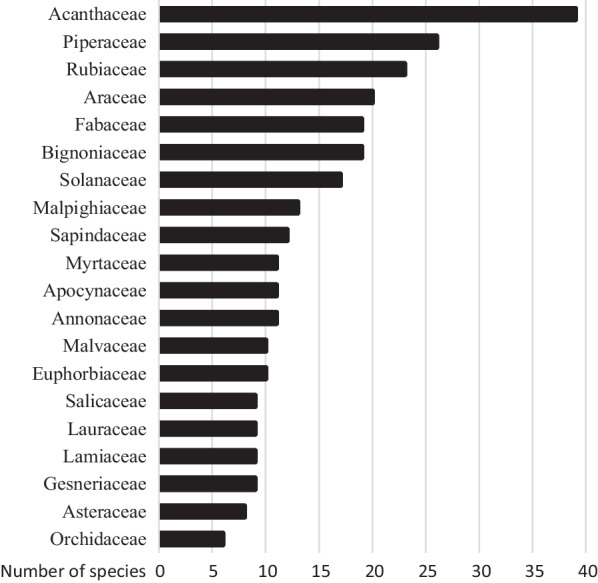
Predominant botanical families. Only the 20 most common families (represented by 6 or more species) are shown

From a total of 467 medicinal taxa collected, there are 99 different botanical families represented in our research, including Acanthaceae, Piperaceae, Rubiaceae, Araceae and Bignoniaceae which together represent more than one quarter of the total number of plants cited (Fig. 2). The situation is similar in other parts of the Ucayali region in which ethnobotanical studies have been carried out among Panoan groups [15, 20].

Representatives of the family Acanthaceae—three species of *Pseuderanthemum*: *P. lanceolatum*—*mikin medan putani bata* were used, mainly for pregnancy and birth disorders, infections manifesting as various forms of herpes and the treatment of upper respiratory tract inflammation. These species, as well as another two species of *Pseuderanthemum*, were used as first aid for venomous snake bites. Another representative of this genus, *xuke bibex bata—P. congestum* (S.Moore) Wassh*.*, was used to treat herpes including shingles and cold sores (12 URs), venomous snake bites (6 UR) and eye problems such as cataracts. *Dunu himi*—*Pseuderanthemum* sp.—was used in 10 cases to treat venomous *Bothrops atrox* bites. Graham [15] also mentions *Aphelandra lasiandra* (Mildbr.) McDade & E.A.Tripp..—*yawan kuxi dau*—as one of the most widely used species in the Acanthaceae family, and this partially coincides with the use of this plant in our research. This plant’s spiny leaves are used to gently whip children’s legs to accelerate their walking. We documented the use of chewed leaves squeezed into the wound of venomous *Scolopendra gigantea* (Amazonian giant centipede) bites, in baths to strengthen a child’s body, in the treatment of headaches, and to treat strong convulsions identified by *vegetalistas*, or shaman, as the attack of a *yawa* (peccary) spirit or “peccary epilepsy”. According to Schultes and Raffauf [46], a toothache rinse can be made from an infusion of this plant. This plant’s root has been found to contain the alkaloid aphelandrine [47].

The Piperaceae family comprises 5 genera, of which *Piper* (about 2,000 species) and *Peperomia* (about 1,600 species) are the most important. The plants grow as herbs, vines, shrubs and trees and are widely distributed and used throughout the tropics and subtropics. Primary forests and forest edges, where we encountered the greatest number of useful species, are home to the greatest variety and abundance of *Piper* plants. Out of a total of 24 *Piper* taxa documented in our research, 14 were identified to species and 10 to genus. The most frequently used were *Piper aduncum* (31 UR), *P. reticulatum* (30 UR), *P. leucophaeum* (22 UR), and *P. marginatum* and *P. peltatum* L. (11 UR each). The Cashinahua people reported using *P. aduncum* leaves to treat digestive problems, pain, headaches, inflammation and fainting, as well as to prevent caries. *P. reticulatum* was primarily used for prenatal or childbirth care and to reduce high fever. Socio-culturally, it was used for behavioural regulation, to lower libido and for hunting. *P. marginatum* was mostly used to alleviate headaches, to cure inflamed teeth and to treat scorpion stings. Out of the 27 *Piper* species reported by Graham [15] there are two congruent species: *yaix maxaka* (*P. nudilimbum*) and *txexen pei* (*P. peltatum*). The first one is used to treat skin blemishes and mycotic infections, which coincides with our research. We documented the use of these two species in treating scabies, skin conditions and skin protuberances in the vagina or anus, genitourinary infections, and as a general restorative. In addition to the common use of *P. peltatum* in treating intestinal parasitosis, other uses such as healing stingray stings, cephalea (headache) and fainting were documented. In an indigenous *Huni Kuĩ* (Kaxinawa) community in the state of Acre, Brazil, adjacent to the current study area, Ehringhaus [13] gathered 48 species of *Piper*, of which 96% were used as analgesics, oral hygiene products and dermatological treatments. The antibacterial and antifungal properties of ethanol extracts of *Piper* spp. leaves and twigs have been examined. Around 44–60% of the extracts inhibited dermatophytic fungal growth, while 69–91% of the extracts inhibited bacterial growth [13]. Terpenoids, alkaloids and phenolic compounds were found in spot tests for secondary plant metabolites. Another genus of Piperaceae was collected, namely *Peperomia*, for which we documented 3 species: *Peperomia blephariphylla* Trel. & Yunck., *Peperomia swartziana* Miq. and *Peperomia pilosa* Ruiz & Pav.

Among the family Rubiaceae (27 spp., 180 UR), the species with the most reported uses was *Uncaria tomentosa* L. with 29 UR. The most frequently documented uses included the treatment of gastritis, rheumatism, internal tumours and hernias. As to the data on the use of plants of the Rubiaceae family in traditional Cashinahua medicine, we agree with Graham [15] regarding the species *kawa* (*Psychotria viridis* Ruiz & Pav*.*), *matsi kawa* (*P. carthagenensis* Jacq*.*), *ixkin tepekan bata* (*Randia armata* (Sw.) DC.) and *xuniwan* (*Geophila macropoda* (Ruiz & Pav.) DC.). The species are identical, but apart from the use of *P. viridis* we report different uses. *R. armata* (25 UR) was used for the treatment of snake bites, respiratory infections and skin diseases. According to Zamora-Martínez and Pola [48], a snake bite is treated in Mexico by taking a mixture of aguardiente (alcoholic liquor) and the fruit and twigs of this plant. Our respondents reported 10 uses of *G. macropoda* to cure inflamed molars and gingival abscesses with mouthwashes mostly in the form of a decoction of leaves. Within the Rubiaceae family, *Psychotria* was the most commonly occurring genus in our study with a wide range of uses. We documented the use of 7 taxa: *P. alba* Ruiz & Pav., *P. carthagenensis*, *P. ruizii* Standl., *P. viridis*, and 3 specimens identified to the genus only. The use of *P. viridis* in the preparation of *nixi pae* (ayahuasca) was recorded by Graham [15]. The leaves of *Psychotria* species are widely recognized as admixtures of the hallucinogenic beverage *ayahuasca* [7]. The leaves of *P. carthagenensis* and *P. viridis* have been found to contain indole alkaloids and sterols [49, 50].

The 20 collected specimens of Araceae which made up part of this study represent at least seven genera and 20 species. Among them, the most prevalent genus was *Philodendron* with 9 species, the most frequent being *P. ernestii* Endl. and *P. fibrillosum* Poepp. No literature reports of ethnomedicinal use, biological activity testing or phytochemical analysis were found for this species.

The most predominant species of Bignoniaceae (169 UR) were *Dolichandra unguis-cati* (L.) L.G.Lohmann, *Cuspidaria floribunda* (DC.) A.H.Gentry, *Tanaecium dichotomum* (Jacq.) Kaehler & L.G.Lohmann, *Jacaranda glabra* (DC.) Bureau & K.Schum. and *Parmentiera cereifera* Seem. For this species, there are no pharmacological reports in the scientific literature, apart from that of Gachet et al. [51], who demonstrate the promising activity of *J. glabra* against the *Plasmodium falciparum* K1 strain.

The species most collected and used from other families were *Leonia glycycarpa* Ruiz & Pav. (Violaceae), *Tradescantia zanonia* (L.) Sw. (Commelinaceae), *Abuta grandifolia* (Mart.) Sandwith (Menispermaceae), *Matisia cordata* Bonpl. (Malvaceae), *Drymonia tenuis* (Benth.) J.L.Clark (Gesneriaceae) and *Mascagnia eggersiana* (Nied.) W.R.Anderson (Malpighiaceae).

We specifically aim to highlight that the selected species in our study, however, do not fully represent the Cashinahua knowledge of medicinal plants.

### Mode of preparation

The predominance of decoctions (1,247 UR), mostly made by boiling plant material in water for topical application (baths, washes and poultices) and for oral ingestion, agrees with that reported in other neighbouring regions [20]. We observed that elderly people generally prefer to prepare their remedies as decoctions because they believe that the more time the plant material is in contact with water, the more effective it is. Our respondents reported several preparation methods including soaking the fresh plant material (usually leaves) in a large volume of cold water (771 UR), soaking ground/pounded/grated leaves in a small volume of cold water (303 UR), and squeezing fresh leaf juice directly on the affected part (251 UR). In the vast majority of cases, water is the vehicle for almost all oral and topic preparations. Without exception, fresh plant material is always used in the preparation of remedies. Two-hundred and fifty-six UR (8.1% of remedies) were prepared in the form of *patarashca*, the typical Amazonian method of preparing small fish wrapped in the leaves of *mani pui* (*Calathea lutea* (Aubl.) Schult.), lightly roasted near the fire. The same form of preparation is applied by the Cashinahua to slightly crushed fresh leaves, to which the seeds of *maxe* “achiote” (*Bixa orellana* L.) are often added, mostly for the treatment of different skin conditions. The wax covering the under surface of *C. lutea* leaves, known in the Amazon as *cauassú*, may serve as a biological defence against infections and herbivory [52]. We presume that when heated this wax may aid in the therapeutic effects of a remedy prepared in form of *patarashca*.

### Mode of application

It is interesting to note that only about 14% of the remedies were taken orally, with the vast majority (85%) being applied topically, most frequently as a bath. The remaining 1% served as protection against epidemics in the form of a fumigant (Table 1). The significant preponderance of externally applied remedies versus internal administration may seem surprising, but it is not uncommon among Amazonian and Andean ethnic groups [53, 54]. Alexiades [40], in his dissertation on the traditional medicine of the indigenous Amazonian Ese Eja people, mentions that 70% of remedies consisted of external treatments which involved direct contact of the plant tissue with the affected body part. The skin offers an accessible and convenient site for the administration of medications. To this end, the field of transdermal drug delivery, aimed at developing safe and efficacious means of delivering medications through the skin, has garnered much time and investment with the continuous advancement of new and innovative approaches [55]. It should be considered that the conventional use of medications in the form of oral administration must overcome the first pass effect, where the active substance enters the digestive tract and undergoes metabolic changes in the liver, which greatly slows down the onset of action and also alters its effects. Kováčik et al*.* [56] claim that the clinical advantages of transdermal drug delivery over traditional administration methods are numerous. In addition, a transdermal drug delivery system has been accepted as a potential non-invasive route of drug administration [57].

**Table 1:** Modes of application of 467 taxa based on 3,154 Use Reports)

Modes of application	User Reports	% of total UR	Administration
Warm or cold bath	851	27	External
Squeeze on/in the affected part	715	22.7	External
Friction (including whole fruit)	514	16.3	External
Ingestion	444	14	Internal
Wash (including mouth wash)	235	7.6	External
Eye drops	176	5.6	External
Plaster	51	1.6	External
Vaginal douche	48	1.5	External
Burned/Fumigation	38	1.2	External
Introduced in affected part	31	1	External
Poultice	30	1	External
Mouth wash	21	0.7	External

The predominant external application methods were warm and cold baths, which together accounted for a total of 32.7% of external uses. Baths were usually prepared in the form of a decoction or pounded leaves macerated in water. The direct application of ground or chewed leaves squeezed on the affected part (22.7%) was used frequently in the first stage of treatment of venomous bites, herpes and cold sores. Crushed plant material heated in the form of *patarashca* was used for skin and subcutaneous cellular tissue disorders, leishmaniosis, infections, inflammations and injuries. Rubbing with fresh or heated leaves ground in a small amount of water (16.2%) was most often applied to areas of inflammation, swelling and pain, but also as a second stage in the treatment of snake bites, as well as in pregnancy care and childbirth. Washing of the affected part (7.4%) was reported to be useful to relieve rheumatic pain, headache, abscesses, haemorrhoids, haemorrhages and irregular menstruation, as well as to cure flu, diarrhoea, inflammations and skin infections. Poultices and plasters (2.6%) of crushed and mostly heated leaves were applied in cases of lumbago, paralysis of the face, nervous tics, bruises, closed wounds and weaning—prevention of mastitis. A special form of application used by different Amazonian ethnic groups [15, 58] is an ocular administration—the instillation of crushed leaf juice directly into the eye (5.6%) which is used to treat conjunctivitis, sties, affections of sight and headaches. However, its most frequent use was during an episode of fainting, dizziness and strong convulsions (a *yuxin* attack) that healers compared with epilepsy, in addition to socio-cultural and magical uses such as sorcery and “panema”, that is, misfortune in hunting. Another common use was its application before going hunting in order to be able to see animals better in the shadows of the forest.

Oral delivery has the advantages of allowing for pre-determined doses, portability and patient self-administration. For these reasons, the oral route in Western culture remains the most convenient means of delivering medications [59, 60]. Ingestion was applied for a wide range of ailments, mainly for the treatment of digestive, genitourinary and respiratory problems, as well as socio-cultural uses such as behavioural regulation and the *nixi pae* (ayahuasca) ceremony.

### Drug activities

Our documented 467 plant taxa are utilized in a variety of contexts, including the treatment of different ailments, the management of social relations, the enhancement of hunting skills, the promotion of the development of healthy and strong infants, and the regulation of fertility. We agree with Graham [15] that the classification of plants according to their medicinal or non-medicinal uses is an artificial system designed for illustration and analysis and in no way reflects the traditional hierarchical classification system referred to us by the Cashinahua. It must also be acknowledged that the species reported are only the result of sampling to date and do not represent the complete knowledge of the medicinal plants of the Cashinahua (Table 2).

**Table 2:** Main therapeutic use categories (≥ 100 UR) presented according to the descending order of the number of UR

Ailment category	UR	% of total UR	ICF	Species count
Sociocultural uses	398	12.6	0.68	128
Poisonings	311	9.8	0.79	65
Infections/Infestations	292	9.2	0.67	98
Pregnancy/birth disorders	275	8.7	0.80	54
Digestive system disorders	246	7.8	0.65	86
Ill-defined symptoms	201	6.4	0.66	70
Nervous system disorders	192	6.1	0.71	56
Inflammations	178	5.6	0.62	68
Painkillers	178	5.6	0.59	73
Injuries	154	4.9	0.70	46
Genitourinary system disorders	141	4.5	0.62	54
Unspecified disorders	113	3.6	0.61	45

UR = Use Report, ICF = Informant Consensus Factor, Species count = overall number of species used for specific ailment category. Note that taxon may be (and usually is) reported in more than one category

The highest number of UR was recorded for socio-cultural uses (398), followed by poisonings (311), infections/infestations (292) and pregnancy/birth disorders (275) as shown in Table 2. The most often quoted health disorders are presented in Table 3, together with the most widespread plant species used to treat them.

**Table 3:** Ten most frequently reported health problems and species with highest citation frequency for their treatment

Medical condition	UR	Species (family), number of Use Reports
Bite	276	*Solanum thelopodium* (Solanaceae), 15
Fainting	182	*Dolichandra unguis-cati* (Bignoniaceae), 14
Child birth	161	*Piper reticulatum* (Piperaceae), 17
Headache	113	*Lacmellea edulis* (Apocynaceae), 9
Dizziness	109	*Dolichandra unguis-cati* (Bignoniaceae), 12
Seizures/Epilepsy	96	*Strychnos tarapotensis* (Loganiaceae), 7
Diarrhoea	84	*Oxalis leptopodes* (Oxalidaceae), *Sanchezia oblonga* (Acanthaceae), 7
Vomiting	81	*Piper heterophyllum* (Piperaceae), 8
Teeth problems	61	*Geophila macropoda* (Rubiaceae), 9
Fever	57	*Esenbeckia febrifuga* (Rutaceae), 12
Tumour	54	*Ficus gomelleira* (Moraceae), 13
Abscess	53	*Solanum nemorense* (Solanaceae), 9
Sting	53	*Aegiphila cuneata* (Lamiaceae), 8
Bleeding	52	*Hiraea fagifolia* (Malpighiaceae), 8

^*^Only the most favoured species (according to UR) are shown

The majority of uses in our study represent envenomation, resulting particularly from snake bites as well as stings and bites from other venomous animals, including scorpions and spiders. Snake bite envenomation is an important global public health issue, especially in tropical areas, due to their frequency and resulting morbidity and mortality [61]; globally, an estimated 81,000–138,000 people a year die from snake bites. In 2017, snake bites were re-added to the list of neglected tropical diseases by the World Health Organization [62]. Several recent studies have demonstrated that the most affected group is mainly composed of men working in rural areas, and that snake bites occur mainly during the day, most frequently involve the lower limbs and are mostly caused by the *Bothrops* genus, which is also the case in South America [63].

Plants for treating snake bites were always the first to be mentioned to us by the research participants and are considered the most culturally important. Poisonings are imminently life-threatening and encounters with venomous snakes in the study area are common. The most common and dangerous bites come from *Bothrops* spp.—*shanu* and *dunu* or “jergón” in Spanish—and *Lachesis* spp.—*kamux* or “shushupe”. We were provided with a comprehensive three-stage treatment for snake bite by all of the Cashinahua plant experts we talked to. The plant for the first stage—emergence—is determined (35 species) on the basis of the species of reptile, and always administered externally by squeezing the juice of chewed leaves (or leaves mashed with a few drops of water) into the wound every 3–5 min until the patient evacuates, which is considered the moment when the venom is eliminated. The most important plant for the first stage treatment was considered to be *Rosenbergiodendron longiflorum* (Ruiz & Pav.) Fagerl—*besti bata*—that was used for both *shanu* and *kamux*, although the types of venom are different. *Casearia obovalis* Poepp. ex Griseb. and *Mascagnia eggersiana* (Nied.) W.R.Anderson leaves are frequently used in an emergency to treat different Viperidae bites. *Lygodium venustum* Sw. is implemented in the treatment of *Bothrops bilineatus* bites. The leaves of *Mascagnia eggersiana* (Nied.) W.R.Anderson, *Caamembeca spectabilis* (DC.) J.F.B. Pastore, *Solanum sessile* Ruiz & Pav. and *Solanum thelopodium* Sendtn. are used to treat *Lachesis muta* bites. The second stage of treatment, that of inflamed wounds, primarily involves bathing the affected part in an herbal decoction (28 species), accompanied by the ingestion of small doses of the same remedy 3–4 times a day, or rubbing the site with leaves pounded in cold water with the same frequency. The final third phase serves to recover the physical strength of the recuperating patient, and can involve 3 species: *Dracontium spruceanum* (Schott) G.H.Zhu, the corm of which is widely used as a snake bite treatment in the Amazon [64], *Cardiospermum halicacabum* L. and *Solanum mite* Ruiz & Pav. Phytochemical screening has revealed that *C. halicacabum* extract contains glycosides, carbohydrates, flavonoids, phytosterols, phenolic compounds and saponins [65]. During all three phases of treatment, the patient must not move and remain lying in a hammock. According to traditional Cashinahua medicine, the treatment of any venomous snake bite is only successful if strict dietary taboos are obeyed; neither the patient nor his healer must eat cooked cassava throughout the treatment; otherwise, the wound remains inflamed. In case of dietary non-compliance, the inflammation becomes chronic and is treated by rubbing the wound with the leaves of *Lepidagathis ipariaensis* Wassh. pounded in a small amount of fresh water.

In many parts of the world, scorpion sting envenomation is also a serious health issue that is often overlooked. The true incidence of scorpion sting envenoming is not known because many victims do not seek medical attention. However, it has been estimated that there are approximately 1 million stings per year [66]. Autonomic nervous system mediators released by scorpion venom can cause myocardial damage, cardiac arrhythmias, pulmonary oedema, shock, paralysis, muscle spasms and pancreatitis—all of which can be fatal in young children. In conjunction with intensive care support, early administration of antivenom is highly effective. *Nixpu bayai* (*Piper leucophaeum*) was the most widely used species in scorpion bite therapy followed by *nidu buxka matsi* (*Piper marginatum* Jacq) and *nishi bata* (*Bunchosia* sp*.*)*.* Slightly grated leaves of the former species are prepared in the form of *patarashca*, and the warm juice is repeatedly squeezed into the wound.

Of the venomous insect bites, the most widespread are that of the “isula” or *buna* ant and the Brazilian wandering spider or banana spider (*Phoneutria nigriventer*)—*xina xuku*. The isula ant or giant/bullet ant (*Paraponera clavata*) is a species of hymenopteran insect of the family Formicidae and the only living member of the genus *Paraponera*. *Piper leucophaeum* was the most used species in isula and spider bite therapy, along with several Salicaceae taxa such as *Casearia obovalis* Poepp. ex Griseb. and *Lunania parviflora* Spruce ex Benth. The most frequently used species to cure venomous spider bites was *Aegiphila cuneata* Moldenke.

Infections and infestations represented the second most frequently cited category in our study (291 UR), which included different types of herpes, leishmaniosis, conjunctivitis and general infestations. The species with the greatest number of UR in this group were *Pseuderanthemum congestum* (S.Moore) Wassh. (13), *Esenbeckia febrifuga* (A.St.-Hil.) A.Juss. ex Mart. (12), *Pseuderanthemum lanceolatum* (11), *Drymonia tenuis* (Benth.) J.L.Clark and *Piper leucophaeum* (10 each). According to our findings, *Jacaranda glabra* (DC.) Bureau & K.Schum. leaves were used to treat advanced states of leishmaniosis (8 UR). The leaves of *Jacaranda copaia* (Aubl.) D. Don, as well as those of *Piper aduncum*, used by the Yanesha displayed favourable activity against *Plasmodium falciparum* in its chloroquine-resistant strain. *Hyptis capitata* Jacq. leaves were used for the treatment of skin problems, while another species, namely *H. lacustris* A. St.-Hil. ex Benth., displayed interesting leishmanicidal activities in the Yanesha pharmacopoeia [67].

Pregnancy, labour and family planning was the third most frequently mentioned category (274 UR), which was cited mostly by female research participants. The species used during gestation, childbirth and the postpartum period were referred to as women’s plants. From this category, *Matisia cordata* (24 UR), *Piper reticulatum* (23), *Tradescantia zanonia* (20), *Theobroma cacao* L. (19), *Pseuderanthemum lanceolatum* (18), *Quararibea wittii* K.Schum. & Ulbr. (17), *Acalypha diversifolia* Jacq. (10) and *Tradescantia zebrina* Bosse (9) are most commonly used. With the exception of *P. reticulatum*, all these plants contain mucilaginous substances (*bixtun*) and are applied to “increase the phlegm” in the woman’s womb, which is considered conducive to healthy foetal development. These plants are believed to be very useful in facilitating childbirth and for the foetus to grow healthy and strong.

The chopped leaves of various *bixtun* plants are applied during a bath in the river once a week until the time when labour pains begin. During the first months of pregnancy, a pregnant woman begins to treat herself with *Piper reticulatum* L*.*: she drinks a small amount of juice from leaves crushed in a cup of water and rubs her belly with the rest of the grated leaves. A single course every month until the time of delivery ensures that the placenta does not grow too large and that it comes out quickly along with the new-born so that the woman does not suffer from waiting. When contractions begin, the potion made from leaves of *P. reticulatum* crushed in cold water is taken to accelerate childbirth. *Urceolina cyaneosperma* (Meerow) Christenh. & Byng and *Pavonia fruticosa* (Mill.) Fawc. & Rendle are used together to facilitate delivery. Twelve of the documented species are antifertility agents with the predominant species being *Chondrodendron tomentosum* Ruiz & Pav., *Clitoria pozuzoensis* J.F.Macbr., *Faramea multiflora* A.Rich. ex DC. and *Rourea amazonica* Radlk. *Ch. tomentosum* is one of the sources of arrow poison curare and it contains, in addition to highly toxic alkaloids, the medicinally valuable alkaloid tubocurarine [68]. To treat women unable to conceive, or to reverse the effect of previously utilized contraceptive plants, the following species, among others, are used: *Ruellia proxima* Lindau, *Pentagonia amazonica* (Ducke) L.Andersson & Rova and *Urceolina cyaneosperma*.

In the treatment of digestive system disorders, the Piperaceae family predominated. In particular, *Piper aduncum* L., *Piper aequale* Vahl., *Piper costatum* C.DC., *Piper heterophyllum* Ruiz & Pav. and *Piper reticulatum* L. were frequently used for diarrhoea, vomiting, constipation and as oral hygiene products. Other utilized species included *Xylosma tessmannii* Sleumer, *Uncaria tomentosa* (Willd. ex Schult.) DC., *Oxalis leptopodes* G. Don, *Sanchezia oblonga* Ruiz & Pav. and *Adenocalymma impressum* (Rusby) Sandwith. In the context of dental care, it is important to mention the use of several species of *Piper* involved in the *nixpu pima* initiation ritual when youngsters (*txipax*) are introduced to the adult world and for the first time their teeth are blackened with the sap of young shots of *nixpu* (*Piper hispidum* Sw. and/or *Piper leucophaeum* Trel.), which is believed to be the best defence against caries.

### Beliefs and symbols in Cashinahua ethnopharmacology

The use of medicinal plants in the study area is highly ritualized, i.e. associated with various kinds of beliefs or with magical or religious practices, as is likely the case in all rural cultures. One important aspect of Cashinahua ethnobotanical practices is the idea that plants play multiple roles at once: medical, social, cultural, pharmacodynamic and symbolic. These roles are subject to significant spatial and temporal variation, as well as frequent interrelations. Ethnobotanical interactions in all of their facets reveal a great deal about broader ecological and social processes [40]. Cashinahua beliefs and practices regarding health, illness and disease cannot be separated from the parallel spirit world, which closely overlays the visible, physical world [15]. The majority of rural cultures do not hold the view that disease and death have an organic or physical cause. The spirit world is the source of both. Kensinger [16] states that one of the ancestral beliefs with which the Cashinahua explain the world refers to the existence of two fundamental aspects: the visible or material side and the invisible side. The visible world is the domain of human beings and all other living things, while the invisible world is the domain of spirits (*yuxin*) that are impossible to see except in dreams and through hallucinogenic experiences. Informal interviews with healers and their patients revealed a widespread belief in the spiritual and magical origin of diseases. According to different authors [15, 40, 53, 54, 58, 69], the majority of native Amazonian people believe that ancestral wrath, sorcery or an attack by a spirit are the causes of sickness and misfortune. According to Sobiecki [70], they frequently point to strained social relationships. Because specific ailments are perceived only as the result of acts of spiritual agents, the herbalists in our study insisted that we include plants that can be considered “magic” without regard to their status.

Unspecified socio-cultural uses of plants (329 UR) presented in our study were related to enhancing hunting skills, changes in personal odour, behavioural regulation, sexual attraction (“puzanga”), misfortune (“panema”) and sorcery. But the prevalent treatment in this category was to cure symptoms interpreted as a spirit (*yuxin*) attack. Our findings confirm the statement of Graham [15] that if a *yuxin* attack were elevated to the rank of illness, it would constitute the most common category of disease reported for the Cashinahua. It would be easy to interpret this as a folk belief, but when we ask what symptoms it manifests, the research participants describe convulsive seizures, often accompanied by loss of consciousness; the affected person is disoriented, suffers great anxiety and cannot control his basic needs. From the point of view of biomedically defined disease states, one might interpret the symptoms of a spirit attack as indicative of some sort of nervous disorder but would never consider a spirit to be an etiologic agent. According to the patient’s behaviour, Cashinahua healers recognize up to six different types of seizures, not unlike epileptic seizures, characterized by their resemblance to animals whose behaviour is similar that of the patient (*yawa*—peccary, *kapa*—squirrel, *txaxu*—deer, *isu*—spider monkey, *xaka*—frog, *amen*—capybara). Each of these kinds of seizure attack is treated with different plants. Graham [15] states that defining and identifying the category of spirit attack is particularly problematic. Treatments in this category include both the use of plants to treat the aftermath of a spirit attack, whose symptoms range from unconsciousness to catatonia to violent range, and the use of plants to protect someone from a spirit attack. In the latter case, plant juice or a decoction is typically taken internally. Warm baths, the application of plant juice to the eye, or both, are typically used as treatment for spirit attacks. Even though mental health issues and psychosomatic disorders have a significant global impact, Western medicine has made little progress in developing long-term treatments that are both sustainable and efficient. To treat these disorders, many traditional societies use ceremonial treatments and mixtures of medicinal plants, frequently to great effect. In order to develop new medications that treat disorders of the nervous system, it may be possible to identify potential future targets through the information gleaned from commonly used traditional treatments [54].

For religious use, only four taxa were presented by research participants, including *Banisteriopsis caapi* (Spruce ex Griseb.) C.V.Morton bark and the leaves of two *Psychotria* species, namely *P. viridis* and *P. alba*, used together in the preparation of the traditional *nixi pae* (ayahuasca) entheogenic brew. Another plant species that is added to this vision-inducing potion is *Renealmia breviscapa* (Poepp. & Endl.) Poepp. & Endl. Alexiades [40] states that ayahuasca is undoubtedly the most widely employed hallucinogen in western Amazonia. Most, if not all, indigenous cultures use hallucinogenic plants as a fundamental part of their healing practices. They frequently play a role that is significantly greater than that of direct therapeutic and calming methods. Research into the tribe’s medicinal plants must therefore include plants used in rituals [46].

### New or very rarely reported medicinal plants

Out of 467 plants that were documented, a review of publications on WoS revealed 79 species that have not yet been published as medicinal and have not undergone phytochemical analysis. These species with little or no pharmacological documentation in the scientific literature are distributed among 60 genera and 42 botanical families, with Acanthaceae being the most represented family with seven species, followed by Fabaceae (six), and Araceae and Solanaceae (four each). The fact that they include 172 uses for 79 new or very little-known medicinal plant species is especially remarkable; Table 4 lists the vascular plants among them, arranged alphabetically by species.

**Table 4:** Medicinal plants used by Cashinahua (*Huni Kuin*) herbalists in Purus Province, Peruvian Amazon, and previously unreported or very rarely cited for medicinal use or phytochemical analysis

Plant species, voucher specimen, family and life form^*^	Vernacular name(s)^*^	Plant part(s) used	Popular use (indications)	Preparation (administration)^#^	FC^‡^ (n = 20)
*Adiantum poeppigianum* C. Presl Hor 149 PTERIDACEAE Herb	*xantxu xeta nenautsi, dunu buxka nenautsi*	Leaf	Digestive problems Menstrual pains Injuries Inflamed wound Snake bite (*tada kamakia*) Abortifacient	Soaked (I, ingestion) Decoction (E, wash) Chewed up (E, squeeze in the affected part) Ground/pounded (E, plaster) Decoction (E, wash) Ground/pounded (E, squeeze in the affected part), decoction (E, wash) Squeezed (I, ingestion)	1 1 13 12 8 11
*Aegiphila cuneata* Moldenke Hor 140 LAMIACEAE Herb	*kunubin kabia*	Leaf Leaf, stem bark	Leishmaniosis Poisoning (spider bites)	Chewed up material or Patarashca (E, squeeze in the affected part) Pounded (E, squeeze in the affected part)	12 2
*Aphelandra acrensis* Lindau Hor 087 ACANTHACEAE (Sub)shrub	*yame bebe*	Leaf	Dizziness Nightmares Facial palsy	Decoction (E, warm bath) Decoction (E, cold bath) Soaked (E, cold wash)	11 3 14
*Aphelandra caput-medusae* Lindau Hor 079 ACANTHACEAE (Sub)shrub	*basikun bexiwa*	Leaf	Fainting	Decoction (E, warm bath)	10
*Aphelandra lasiandra* (Mildbr.) McDade & E.A.Tripp Hor 112 ACANTHACEAE Shrub	*yawan kuxi dau* *yawan kuxi dau bata* *yawan xuke dau* *txikix payati matsi*	Entire plant Leaves	Muscle stimulant Seizures, epilepsy of *yawa* Venomous bite *awawa*-*Scolopendra gigantea* Headache Insomnia, nightmare	Soaked (E, cold bath) Decoction (E, warm bath) Chewed or pounded (E, direct application) Pounded (E, wash) Soaked (E, wash) Squeezed (E, eye drops)	12 14 2 2 3 2
*Aristolochia odoratissima* L Hor 539 ARISTOLOCHIACEAE Climber	*nai txi wexpa*	Leaves Unspecified Aerial parts	Vomiting Newborn diarrhoea Fainting	Decoction (E, warm bath) Decoction (E, warm bath) Decoction (E, warm bath)	2 2 9
*Asplenium angustum* Sw Hor 116 ASPLENIACEAE Epiphyte	*txaxu kexa*	Leaves	Sores	Patarashca (E, mouth washes, I ingestion)	13
*Asplenium serratum* L Hor 224 ASPLENIACEAE Epiphyte	*nuntu tae*	Leaves	Gallbladder disorders Gallbladder, cirrhosis Tumour Pain Canker sores	Heated up (E, cataplasm) Heated up (E, cataplasm) Heated up (E, cataplasm) Heated up (E, cataplasm) Patarashca* (E, squeeze in the affected part),	2 3 3 9 1
*Begonia maynensis A. DC* Hor 170 BEGONIACEAE Herb	*tetun pei matsi taxipa*	Leaves	Treats all diseases Upset stomach Gallbladder, liver Ovary, kidney Hyperthermia Chills, tremor Facial palsy Snake bite *shanu* Bronquitis	Soaked (I, ingestion) Soaked (I, ingestion) Soaked (I, ingestion) Decoction (E, warm bath) Soaked (E, cold bath) Decoction (E, warm bath) Soaked (E, friction) Soaked (E, friction) Soaked (I, ingestion)	1 4 2 2 2 3 1 5 10
*Bomarea edulis* (Tussac) Herb Hor 162 ALSTROEMERIACEAE Herb	*dei yuxibun bixtu bexea*	Leaves	Tranquilliser Facial palsy	Decoction (E, warm bath) Soaked (E, local wash, friction)	4 18
*Casearia obovalis* Poepp. ex Griseb Hor 249 SALICACEAE Shrub/tree	*xipintun akai bata*	Leaves Stem bark	Boils Groin hernia Venomous spider bites Snake bites *shanu*	Heated up (E, cataplasm) Soaked (E, direct application) Chewed (E, direct application) Chewed (E, direct application), Soaked (E, friction), decoction (E, warm bath)	1 1 2 9
*Centropogon cornutus* (L.) Druce Hor 377 CAMPANULACEAE (Sub)shrub	*isku xeta bata* *xudi batxia*	Leaves	Lymphatic disorder Canker sores, cold sores	Heated up (E, cataplasm), decoction (E, warm bath) Patarashca (E, direct application)	4 15
*Clavija nutans* (Vell.) B.Ståhl Hor 151 PRIMULACEAE Shrub	*maspanewan*	Leaves	Black diarrhoea Influenza Infected throat	Decoction (E, warm bath) Soaked (I, ingestion) Pounded (E, direct application)	9 9 1
*Clavija weberbaueri* Mez Hor 111 PRIMULACEAE Shrub/tree	*maspanewan*	Leaves	Black diarrhoea Hernia Testicle descended, fever Infected throat Boils	Decoction (E, warm bath) Soaked (E, friction) Soaked (E, friction) Soaked or heated up (I, ingestion) Pounded (E, direct application)	13 2 6 13 2
*Clitoria amazonum* Mart. ex Benth Hor 433 FABACEAE Shrub/tree	*nenautsi xankuma*	Leaves	Menstrual pain Snake bite *shanu* Postpartum disorders Permanent contraception	Decoction (E, vaginal douche) Squeezed (E, direct application), decoction (E, wash) Decoction (E, warm bath) Decoction (E, warm bath, vaginal douche)	1 12 2 2
*Clitoria pozuzoensis* J. F.Macbr Hor 432 FABACEAE Shrub/climber	*nenautsi himiya* *tene kabia nenautsi*	Leaves Stem bark Roots	Joint and muscle pain Long lasting contraception	Decoction (I, ingestion), (E, warm bath) Decoction (I, ingestion), (E, warm bath, (E, vaginal douche), squeezed (E, eye drops)	10 9
*Connarus punctatus* Planch Hor 199 CONNARACEAE liana	*anu xaxe*	Leaves Leaves and bark	Seizures, body tremor Conjunctivitis Cracked skin of the foot	Decoction (E, warm bath) Soaked (E, wash, eye drops) Patarashca (E, squeeze in affected part)	8 14 1
*Cordia nodosa* Lam Hor 446 BORAGINACEAE Shrub/tree	*kapa yubu*	Leaves Leaves and bark	Testicular inflammation Seizures, epilepsy Venomous bite—spider	Soaked (E, direct application) Squeezed (E, eye drops) Squeezed (E, direct application)	1 12 11
*Cuspidaria floribunda* (DC). A. H Gentry Hor 177 BIGNONIACEAE Liana/climber	*hima nuin*	Leaves	Herpes zoster, shingles Boils Allergic dermatosis	Patarashca or soaked (E, squeeze in affected part) Patarashca (E, direct application) Soaked (E, direct application)	15 3 2
*Desmodium axillare* (Sw.) DC Hor 054 FABACEAE (Sub)shrub	*xanu tamu nenautsi*	Leaves	Muscle stimulant	Decoction (E, warm bath, wash)	12
*Dioscorea acanthogene* Rusby Hor 243 DIOSCOREACEAE Climber	*dantan ikan hina*	Leaves	Joint and body pain, rheumatism	Decoction (E, warm bath, wash)	10
*Dolichandra uncata* (Andrews) L.G. Lohmann Hor 213 BIGNONIACEAE Liana/climber	*bunpa mentsisa*	Leaves	Pain in the ribs and body Dizziness, loss of consciousness	Decoction (E, warm bath) Squeezed (E, eye drops) Decoction (E, warm bath)	10 6
*Drymonia coccinea* (Aubl.) Wiehler Hor 064 GESNERIACEAE Epiphyte/climber	*xuke txixin bata*	Leaves	Haemorrhoids Testicular inflammation	Soaked (E, direct application) Soaked (E, direct application), pounded (E, friction)	2 14
*Drymonia tenuis* (Benth.) J. L. Clark Hor 189 GESNERIACEAE Epiphyte/climber	*nuin hene watima*	Leaves Leaf juice	Cutaneous infection Conjunctivitis Nightmares, tranquilliser Herpes zoster Stye Sight disorder	Patarashca (E, direct application) Soaked (E, eye drops) Squeezed (E, eye drops) Patarashca (E, direct application) Squeezed (E, eye drops) Squeezed (E, eye drops)	2 6 4 4 2 2
*Eirmocephala brachiata* (Benth. Oerst.) H. Rob Hor 543 ASTERACEAE (Sub)shrub	*kape txinkan*	Leaves	Muscle relaxant Lumbar spine pain	Decoction (E, warm bath) Heated up (E, cataplasm) Decoction (E, wash)	9 5 5
*Erythrina ulei* Harms Hor 023 FABACEAE Tree	*kaxu* *amasisa* (Spanish)	Leaves Stem bark	Infections in general Inflamed wound	Decoction (E, warm bath), Soaked (E, wash) Pounded (E, friction)	11 10
*Fischeria stellata* (Vell.) E. Fourn Hor 101 APOCYNACEAE Climber	*yawa tsis nuin*	Leaves	Ear inflammation Open wounds Skin infection	Patarashca (E, direct application) Patarashca (E, direct application) Patarashca (E, direct application)	2 10 11
*Fridericia japurensis* (DC.) L. G.Lohmann Hor 204 BIGNONIACEAE Liana	*nuin himi taseya*	Leaves	Lymph glands disorders Lymphogranuloma Boils	Soaked (E, friction) Soaked (E, friction) Pounded (E, friction), soaked (E, squeeze in the affected part)	2 2 12
*Goeppertia pavonii* (Körn.) Borchs. & S.Suárez Hor 167 MARANTHACEAE Herb	*mani pei taxipa xiwaya*	Leaves	Vomit, diarrhoea Fainting emergency Fainting Nightmares, insomnia Convulsions, epilepsy Headache	Squeezed (E, eye drops) Squeezed (E, eye drops) Decoction (E, warm bath) Squeezed (E, eye drops) Decoction (E, warm bath) Squeezed (E, eye drops)	1 2 2 1 10 1
*Guazuma crinita* Mart Hor 191 MALVACEAE Tree	*patxa kaman kenan* *bolaina blanca* (Spanish)	Leaves	Behaviour disturbances Sting ray Scabies	Decoction (E, warm bath) Patarashca (E, direct application) Patarashca (E, direct application)	18 1 9
*Herrania balaensis* P. Preuss Hor 225 MALVACEAE Tree	*nesan paubin*	Leaves	Tranquilliser Stiff neck Lumbago	Decoction (E, warm bath) Heated up (E, cataplasm) Heated up (E, cataplasm)	1 2 2
*Hymenopus arachnoideus (Fanshawe & Maguire) Sothers & Prance* Hor 214 CHRYSOBALANACEAE Tree	*nixu pei dani uma nia*	Leaves	Facial palsy Fainting, insanity	Soaked (E, friction) Decoction (E, warm bath)	1 10
*Justicia dumetorum* Morong Hor 472 ACANTHACEAE (Sub)shrub	*matsi dantunkuya*	Leaves	Chill without fever, hypothermia	Decoction (E, warm bath)	12
*Lacistema aggregatum* (P. J. Bergius) Rusby Hor 176 LACISTEMATACEAE Shrub/tree	*xane tenan metxa*	Leaves	Protuberance in vagina Prolapse Boils Strong headache Labour induction Postpartum headache	Patarashca (E, direct application) Pounded (E, direct application) Soaked (E, squeeze directly) Soaked (E, wash), decoction (E, wash), Patarashca* (E, squeeze in the affected part) Soaked (E, friction) Decoction (E, warm bath)	10 1 1 8 1 1
*Lacmellea edulis* H. Karst Hor 181 APOCYNACEAE Tree	*hane bata*	Leaves	General weakness Vomiting, dizziness, nausea Headache Headache, fainting	Pounded (E, friction) Soaked (I, ingestion) Soaked or heated up (E friction) Decoction (E, warm bath)	2 2 3 6
*Leonia glycycarpa* Ruiz & Pav Hor 391 VIOLACEAE Tree	*tunku dau bata*	Leaves Stem bark	Diarrhoea due to infection Struma Boils Inner tumour External tumour Shoulder or hip pain Snake bite *shanu, kamux*	Decoction (I, ingestion) Decoction (E, friction) Soaked (E, direct application) Decoction (I, ingestion) Decoction, soaked (E, friction) Decoction (E, friction) Pounded (E, squeezed in wound)	1 2 2 2 8 1 14
*Machaerium cuspidatum* Kuhlm. & Hoehne Hor 122 FABACEAE Liana	*kapa xeta nenautsi*	Leaves	Open wound, cut Body pain Venomous bite *mai dunu* Skin affections	Chewed, pounded, heated up (E, direct application) Decoction (E, warm bath) Soaked (E, squeezed in wound) Patarashca (E, squeeze in affected part), decoction (E, wash)	20 1 1 2
*Manihot brachyloba* Müll. Arg Hor 493 EUPHORBIACEAE Shrub/climber	*dua pei*	Leaves	Dizziness, fainting Headache	Decoction (E, warm bath) Decoction (E, wash)	10 10
*Mascagnia eggersiana* (Nied.) W.R.Anderson Hor 172 MALPIGHIACEAE Climber	*nixi bata pei txumi*	Leaves	Inflamed tooth, swelling Snake bite *shanu*	Decoction (E, plaster), pounded (E, squeezed in the mouth) Pounded, soaked (E, squeezed in wound)	7 19
*Mayna odorata* Aubl Hor 067 ACHARIACEAE Shrub/tree	*date maxan* *maxanewan* *maxamawan*	Leaves	Testicular inflammation Tinea capitis Epilepsy Headache Skin affections Postpartum disorders	Soaked, pounded (E, wash) Patarashca (E, direct application) Decoction (E, cold bath) Decoction (E, warm bath) Patarashca (E, direct application) Soaked (I, ingestion)	2 1 1 9 9 2
*Mendoncia pedunculata* Leonard Hor 086 ACANTHACEAE Liana	*bunpa pei xiwaya*	Leaves	Epilepsy, seizures Body pain Snake bite *shanu* Otitis	Decoction (E, warm bath) Soaked (E, wash) Chewed (E, squeeze in wound) Patarashca (E, direct application)	10 10 10 1
*Myrcia densiflora* (Poepp. ex O. Berg) A. R.Lourenço & E. Lucas Hor 093 MYRTACEAE Shrub/tree	*mani yuxin*	Leaves	Dizziness, nightmares Fainting	Decoction (E, cold bath) Pounded (I, squeeze the leaf juice in the mouth)	1 10
*Myrcia lonchophylla* A. R.Lourenço & E. Lucas Hor 136 MYRTACEAE Shrub/tree	*kankan takanpi*	Leaves	Vomit, diarrhoea, fainting	Soaked (I, ingestion), decoction (E, warm bath)	12
*Matisia cordata* Bonpl Hor 422 MALVACEAE Tree	*ixtxibin* *sapote* (Spanish)	Leaves	Labour induction Pregnancy care	Soaked (I, ingestion), pounded (E, friction, wash) Soaked (E, friction, wash)	14 10
*Nautilocalyx pallidus* (Sprague) Sprague Hor 280 GESNERIACEAE Herb	*txatxa matsi* *awa himi xudu dau*	Leaves	Fever or flu prolonged Gallbladder inflammation Hyperthermia Bone and joint pain Seizures, epilepsy Body paralysis	Decoction (E, warm bath), soaked (E, friction) Decoction (E, wash) Soaked (E, cold bath) Soaked (E, friction) Squeezed (E, eye drops) Soaked (E, cold bath)	4 2 1 2 2 1
*Neea divaricata* Poepp. & Endl Hor 042 NYCTAGINACEAE Shrub/tree	*kuxun himi* *txuxtiwan* *txuxti*	Leaves	Bleeding	Decoction (E, warm bath)	15
*Neea spruceana* Heimerl Hor 096 NYCTAGINACEAE Shrub/tree	*txuxtiwan*	Leaves	Flatulence Bleeding Snake bite *mai dunu*	Pounded (E, poultice) Decoction (E, warm bath) Soaked, Patarashca (E, squeezed in the wound)	10 1 4
*Oxalis leptopodes* G. Don Hor 232 OXALIDACEAE Sub/shrub	*tete bexmi*	Leaves	Chronic diarrhoea Strong diarrhoea, vomit	Decoction (E, wash, friction) Decoction (I, ingestion)	10 6
*Passiflora araujoi* Sacco Hor 091 PASSIFLORACEAE Climber	*nai tatxa*	Leaves	Restorative, vital tonic Fainting, dizziness	Decoction (E, warm bath) Decoction (E, cold bath)	10 1
*Paullinia tenera* Poepp. & Endl Hor 016 SAPINDACEAE Climber	*hasim punu nenautsi*	Leaves	Inflammation after snake bite Body pain after hard work Twisted joint	Decoction (E, warm bath) Decoction (E, warm bath) Decoction (E, warm bath)	2 2 10
*Pentagonia amazonica* (Ducke) L. Andersson & Rova Hor 104 RUBIACEAE Tree	*nanewan*	Leaves and bark Fruits Leaves	Epilepsy, seizures Increases fertility Increases fertility Dizzy, fainting, seems insane	Decoction (E, warm bath) Unprocessed (I, ingestion) Decoction (E, cold bath) Decoction (E, cold bath)	13 1 1 1
*Philodendron ernestii* Engl Hor 218 ARACEAE Epiphyte/climber	*xuni pei tatxunya*	Leaves Unspecified aerial parts	Nervous tic Lumbar spine pain Facial palsy	Heated up (E, cataplasm), pounded (E, direct application) Heated up (E, friction) Decoction (E, friction, wash, poultice)	16 4 4
*Philodendron exile* G. S. Bunting Hor 100 ARACEAE Epiphyte/climber	*baxu taka nixi* *upi dau pei mesi*	Leaves	Vomit	Soaked (I, ingestion)	11
*Philodendron fibrillosum* Poepp Hor 540 ARACEAE Epiphyte/climber	*in tabi*	Leaves	Pregnancy protection Labour induction Boils	Soaked (I, ingestion), (E. friction) Soaked (I, ingestion), (E, friction) Soaked (E, wash)	3 4 10
*Philodendron toshibae* M. L. Soares & Mayo Hor 110 ARACEAE Epiphyte/climber	*xawe batxi nuin* *xuni pei keneya*	Leaves	Female urinary infection Tumour	Decoction (E, wash) Leaf juice (E, friction)	10 1
*Piparea multiflora* C. F.Gaertn Hor 184 SALICACEAE Shrub/tree	*inu kexni*	Leaves	Gastrointestinal disorder Strong constipation, Dysentery	Decoction (E, wash) Soaked (I, ingestion) Decoction (E, warm bath)	10 6
*Piper casapiense* (Miq.) C. DC Hor 107 PIPERACEAE Shrub	*awa denpan nixpu*	Leaves	Respiratory problems, swollen nose	Pounded (E, friction)	11
*Piper costatum* C. DC Hor 088 PIPERACEAE Shrub	*babu dau matsi*	Leaves	Dental follicle Inflamed tooth	Pounded (E, squeeze in the mouth) Grated (E, introduce in the teeth)	11 2
*Piper leucophaeum* Trel Hor 139 PIPERACEAE Shrub	*nixpu bayai*	Leaves	Snake bite *pexie xeta* Tooth protection *Nixpu pima* ceremony	Pounded (E, squeeze in the wound), decoction (E, warm bath) Unprocessed twigs (E, friction)	2 11
*Pristimera tenuiflora* (Mart. ex Peyr.) A. C. Sm Hor 142 CELASTRACEAE Liana	*nixi metunya*	Leaves	Any inflammation Snake bite *shanu pexie xeta*	Decoction (E, warm bath) Decoction (E, warm bath)	10 2
*Prunus myrtifolia* (L.) Urb Hor 158 ROSACEAE Tree	*biunx haxu*	Leaves	Genitourinary infection Persistent fever Snake bite *kana dunu*	Decoction (E, wash), patarashca (E, direct application) Decoction (E, cold bath) Pounded (E, squeezed in the wound)	10 2 2
*Pseuderanthemum lanceolatum* (Ruiz & Pav.) Wassh Hor 412 ACANTHACEAE Herb	*mikin medan putani bata*	Leaves	Herpes, mycosis Estomatitis herpetica Always tears the eye Epilepsy, seizures Pimples in the mouth Pregnancy protection Labour induction Snake bite	Decoction (E, direct application) Chewed (E, direct application) Soaked (E, wash) Decoction (E, bath) Patarashca (E, squeeze in the mouth) Soaked (E, friction) Soaked (E, friction), (I, ingestion) Grounded (E, squeeze in the wound)	2 1 2 1 6 2 5 2
*Pulchranthus adenostachyus* (Lindau) V. M Baum, Reveal & Nowicke Hor 005 ACANTHACEAE Herb	*xuke bibex bata pei ewapabu*	Leaves	Venomous bite *xukedun* Snake bite *kana dunu* Snake bite *menpax* Cold sores Eye disorders Herpes	Soaked (E, squeeze in the wound) Grounded (E, squeeze in the wound) Grounded (E, squeeze in the wound) Patarashca (E, squeezed in the mouth) Chewed (E, direct application)	1 1 2 2 1
*Quararibea wittii* K.Schum. & Ulbr Hor 431 MALVACEAE Tree	*tui pei*	Leaves	Labour induction, cervical dilator Pregnancy care Newborn protection	Soaked (I, ingestion), pounded (E, friction, wash) Soaked (E) friction, wash Decoction (E, warm bath)	12 5 2
*Rhynchospora umbraticola* Poepp. & Kunth Hor 097 CYPERACEAE Herb	*kamanen xatxi*	Leaves	Bowel infection Dog bite Rheumatism, arthritis	Decoction (E, wash) Decoction (E, warm bath) Decoction (E, wash, friction)	10 2 11
*Rosenbergiodendron longiflorum* (Ruiz & Pav.) Fagerl Hor 004 RUBIACEAE Tree	*besti bata*	Leaves	Skin infection Snake bite	Patarashca (E, direct application) Chewed or pounded (E, squeeze in the wound)	1 17
*Rourea amazonica* (Baker) Radlk Hor 287 CONNARACEAE Shrub/climber	*nenautsi himia* *tenekabia nenautsi*	Leaves	Menorrhagia Any inflammation Deep cuts Cancer Body pain Postpartum disorders Long lasting contraceptive	Decoction (E, warm bath) Decoction (I, ingestion) Decoction (I, ingestion), (E, squeeze in the wound) Decoction (I, ingestion) Decoction (E, warm bath) Decoction (E, wash) Decoction (I, ingestion)	4 2 3 2 2 1 2
*Ruizodendron ovale* (Ruiz & Pav.) R. E. Fr Hor 061 ANNONACEAE Tree	*kudu xai*	Leafy branches	Protection against epidemic	Burned (E, fumigant)	12
*Schnella hirsutissima* (Wunderlin) Trethowan & R. Clark Hor 246 FABACEAE Shrub/climber	*nixi pei dania* *awa benen be pasa nixi*	Leaves Entire plant ex situ	Malaise-fatigue, anxiety Fainting Haemorrhage Blurred view	Squeezed (E, eye drops) Decoction (E, warm bath) Decoction (E, warm bath) Heated up (E, local wash)	5 5 2 2
*Siparuna cervicornis* Perkins Hor 124 SIPARUNACEAE Shrub/tree	*yuxin bia*	Leaves	Shock symptoms Insanity, fainting, anxiety	Decoction (E, warm bath) Decoction (E, cold bath)	10 1
*Solanum anceps* Ruiz & Pav Hor 319 SOLANACEAE Shrub	*utsi bata pei taxipa*	Leaves	Ovarian inflammation Boils Inflamed wound after the snake bite	Decoction (E, vaginal douche) Soaked (E, squeezed) Soaked (E, squeezed in the affected part)	4 3 1
*Solanum barbeyanum* Huber Hor 439 SOLANACEAE Shrub/climber	*i txiux*	Leaves Fruit	Mastitis Boils Infected wound Boils prevention	Soaked (E, friction, squeezed in affected part) Pounded (E, wash) Pounded, soaked (E, wash) Unprocessed (I, ingestion)	2 6 9 3
*Solanum sessile* Ruiz & Pav Hor 010 SOLANACEAE Shrub	*xau bata*	Stem bark Leaves	Hyperhidrosis Bone and joint pain Snake bite *kamux*	Soaked (E, cold bath) Decoction (E, warm bath) Patarashca (E, squeezed in the affected part), soaked (E, friction), decoction (E, warm bath)	13 3 20
*Solanum thelopodium* Sendtn Hor 303 SOLANACEAE Shrub	*kamuxun bata*	Leaves	Snake bite *kamux*	Chewed or pounded (E, squeezed in the wound), soaked (E, friction)	15
*Strychnos tarapotensis* Sprague & Sandwith Hor 013 LOGANIACEAE Climber	*nutxun tun*	Leaves	Facial palsy Convulsions, epilepsy “susto”	Pounded (E, wash), infusion (E, wash) Decoction (E, warm bath) Infusion (E, warm bath)	6 7 1
*Tanaecium dichotomum* (Jacq.) Kaehler & L. G. Lohmann Hor 163 BIGNONIACEAE Liana	*inawan madi itsa*	Leaves	Fainting Epidemic protection Newborn cries to faint Convulsions, epilepsy Disease protection	Decoction (E, warm bath) Burned (E, fumigant) Decoction (E, warm bath) Decoction (E, warm bath) Decoction (E, warm bath)	10 4 4 4 5
*Tradescantia zanonia* (L.) Sw Hor 317 COMMELINACEAE (Sub)shrub	*bake bixtun* *txaxu bake bixtun cañagua* (Spanish)	Leaves	Epilepsy, seizures Pregnancy care Labour induction	Decoction (E, cold or warm bath), squeezed (E, eye drops) Soaked (E, friction) Soaked (I, ingestion), (E, friction)	5 5 20
*Urceolina cyaneosperma* (Meerow) Christenh. & Byng Hor 105 AMARYLLIDACEAE Herb	*dunu huda* *anu maspu*	Entire plant ex situ	Pain in the legs Labour induction	Pounded (E, friction) Soaked (I, ingestion), pounded (E, friction)	1 16
*Xylosma tessmanii* Sleumer Hor 223 SALICACEAE Shrub/tree	*inu kexni*	Leaves	Gastrointestinal infection Constipation, stomach pain, colic	Decoction (E, wash) Decoction (E, warm bath), soaked (I, ingestion)	10 6

*Species names according to the Plants of the World Online (POWO 2023), family classifications follows APG IV (Byng 2019). Local names in *Hantxa Kuin* if not stated otherwise; # E: external use, I: internal use ‡ FC: Frequency of citation

In Table 4, taxa with a frequency of citation (FC) higher than ten are potential candidates for phytochemical or pharmacological research. The following is a discussion of some other notable species in addition to the data presented in Table 2. A wide variety of applications characterize a few species, and five of them stand out because of their adaptability: *Aphelandra lasiandra* (Mildbr.) McDade & E.A.Tripp., *Rourea amazonica* (Baker) Radlk., *Adiantum poeppigianum* C. Presl, *Begonia maynensis* A. DC. and *Leonia glycycarpa* Ruiz & Pav.

Of the 79 highlighted species in this research, 11% (12 species) were also described by Manduca et al*.* [14], who discussed the traditional medicine of the Brazilian *Huni Kuĩ* (Kaxinawa), with some variations in phonetics and spelling in *Hantxa Kuin*; 6 species present the same taxonomy, with *Leonia glycycarpa* Ruiz & Pav., *Clavija weberbaueri* Mez, *Nautilocalyx pallidus* (Sprague) Sprague, *Aphelandra acrensis* Lindau, *Cordia nodosa* Lam. and *Prunus myrtifolia* (L.) Urb. being the most frequently mentioned.

Some of the taxa reported in Table 4 have the same uses as other species of the same genus; this is the case for *Quararibea wittii* K.Schum. & Ulbr. and *Q. guianensis* Aubl., *Drymonia coccinea* (Aubl.) Wiehler and *Drymonia semicordata* (Poepp.) Wiehler, and *Paullinia tenera* Poepp. & Endl*.* and *Paullinia* sp., among others. Similarly, Graham [15] mentioned 19% of the species found in our study in his dissertation thesis, in which 7% (4 species) present taxonomic correspondence of genera and species, with similar local names, while he mentioned 14% (8 species) with the same genus but other species and different local names.

Among the plants used for curing inflammations, *Forsteronia graciloides* Woodson, cited by six study participants as the universal plant for curing all types of this frequent disorder, is worth mentioning. Strong antiherpetic activity is attributed to 21 species, among which the most cited are *Pseuderanthemum congestum*, *P. lanceolatum*, *Caamembeca gigantea* (Chodat) J.F.B. Pastore and *C. spectabilis* (DC.) J.F.B. Pastore. We did not find any previous reports on medicinal uses for *P. congestum*, *P. lanceolatum* and *C. gigantea* whereas *C. spectabilis* is only cited in Brazil in the community of Caruarú [71]. These medicinal plants, very rarely cited, also deserve further investigation.

We also cross-checked our 79 species with the World Checklist of Useful Plant Species, which includes 40,292 plant species [72], and found that none of the four *Philodendron* spp. and three *Piper* spp. from our study have been reported previously. Remarkably, even though *Solanum* was the most cited “useful genus” (with 328 species) in the checklist, none of the four species included in this paper have been reported before for any medicinal usage.

Also, only 5 out of the 79 species in this study (*Cordia nodosa* Lam., *Centropogon cornutus* (L.) Druce, *Tradescantia zanonia* (L.) Sw., *Desmodium axillare* (Sw.) DC. and *Leonia glycycarpa* Ruiz & Pav.) are included in the list of more than a thousand medicinal plants used in the Peruvian Amazon published by the *Instituto De Investigaciones De La Amazonia Peruana* (IIAP) [73].

### Comparative study of medicinal plant species and genera

**Fig. 3:**
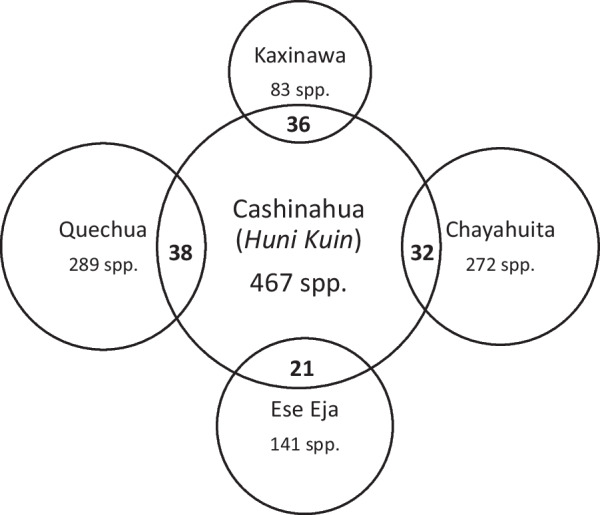
Venn diagram comparing the number of medicinal plant taxa documented in the present study and studies of other Amazonian ethnic groups

The overlap between the cultures is illustrated through a Venn diagram (Fig. 3), and Jaccard’s similarity indices are shown in Table 5.

**Table 5:** Comparison of medicinal plant species documented in Purus Province and neighbouring regions based on available ethnobotanical studies

Region and administrative department	Ethnic group	No. of genera	No. of species	No. of identical genera	No. of identical species	Jaccard Index (genera)	Jaccard Index (species)	Reference
Brazilian Amazon Jordão River, State Acre	*Kaxinawa* (*Huni Kuĩ*)	72	83	56	36	20.82	8.89	Penedo et al 2023
Peruvian Amazon Chazuta Valley, San Martín	*Quechua* *(Lamas Quechuas)*	202	289	92	38	25.34	6.24	Sans-Bizet *et. al* 2008
Peruvian Amazon Paranapura Basin, Loreto	*Chayahuita* *(Shawi)*	191	215	74	32	20	5.91	Odonne *et. al* 2013
Bolivian Amazon, La Paz Peruvian Amazon, Madre de Dios	*Ese Eja* *(Huarayo)*	123	129	46	21	13.93	4.51	Alexiades 1999
Peruvian Amazon Curanja River, Ucayali	*Cashinahua* *(Huni Kuin)*	253	358	N/A	N/A	N/A	N/A	The present study
Peru—all country All departments	Indigenous and mestizo cultures	571	1028	149	75	22.07	5.72	IIAP 2010

Not surprisingly, the highest degree of similarity at the genus and species level was determined for medicinal plants used by the Brazilian Kaxinawa (*Huni Kuĩ)* of the Jordao River area, in the neighbouring state of Acre, although the number of documented taxa is considerably lower. Notable commonalities were detected with regions in other parts of the Peruvian Amazon. The lowest level of similarity was found with a study on traditional medicine of the Ese Eja people inhabiting both banks of the Heath River, which forms the border between Peru and Bolivia.

So far, the largest number of medicinal species used has been documented in the Alto Purus River basin (Department of Ucayali and State of Acre) with 411 species (this study and [41]), followed by the Chazuta Valley (Department of San Martín) with 289 species [42], the Paranapura Basin (Department of Loreto) with 215 species [43], the Heath River basin in the Peruvian Department of Madre de Dios and the neighbouring Bolivian Department of La Paz (129 species [40]). The only taxon that is consistent across all of the compared studies is *Petiveria alliacea* L. The following are medicinal species that correspond to at least three of the four studies: *Abuta grandifolia* (Mart.) Sandwith, *Banisteriopsis caapi* (Spruce ex Griseb.) C.V.Morton, *Bixa orellana* L., *Calycophyllum spruceanum* (Benth.) K.Schum., *Dracontium spruceanum* (Schott) G.H.Zhu and *Mansoa alliacea* (Lam.) A.H.Gentry.

From a total of 96 taxa that showed correspondence with medicinal species recorded in our study, we discovered approximately 270 novel uses with a therapeutic purpose and 27 new uses related to culture. These novel uses are beyond the scope of this publication and will therefore be the subject of the subsequent article.

### Medicinal plant use versus conservation

The province of Purus is considered one of the biodiversity hotspots for conservation priorities [74]. Culturally undisturbed regions such as the Curanja River area continue to harbour considerable biocultural richness. In this region and other parts of the world, plant use and conservation may conflict. Some plant species may suffer from high collection pressure for medicinal purposes, as has been noted in various nations [75, 76, 77, 78, 79]. But the Cashinahua, as well as other Amazonian ethnic groups, who live in the world’s largest basin in terms of biodiversity and water reserves, are true conservationists. Because they only consume daytime plants and animals, they profit from the flora and fauna without destroying it, with complete respect for the natural environment. There is no place for species accumulation or indiscriminate exploitation, as this would violate the principles governing human interaction with nature. The cultural wealth that enables these peoples to live in harmony with nature is known as ancestral knowledge. Another, arguably positive, aspect of the conservation issue is the remoteness and inaccessibility of the province, especially the upper reaches of the Curanja River, which significantly limits the presence of outsiders; so medicinal herbs are collected only for use among local patients. Because of the very low population density in the province and dense primary forest that surrounds the communities, it is unlikely that collecting wild herbs would threaten their occurrence. Therefore, harvesting the leaves of medicinal plants never causes conservation concern. An exception is the small percentage of plants whose roots are used, like that of *Dracontium* spp. Among these taxa, highly valued are *Zamia ulei* tubers used for general recovery of the body after a long illness. However, a more popular use of this plant is to cure erectile function and stimulate the libido, as is the use of a decoction of *Abuta grandifolia* leaves. These two plants are the only ones we have recorded as being traded, as they are sent in small quantities to Pucallpa for sale. Otherwise, as far as we know, no other plants are collected for retail. *Manihot brachyloba* (IUCN Red List), used to treat headaches, fainting and dizziness, is often transplanted along the edges of manioc fields (“chacras”) for its protection, causing it to proliferate rather than threatening it.

### Public health significance of the present study

Table 3 lists the most common health conditions in the researched area. Medicinal plants used to treat all these health problems are widely represented in our study.

Envenoming from poisonous animal bites is a serious public health issue in Latin America. Recognizing the impact of poisonings on vulnerable population groups, we would like to emphasize the importance of the species used to treat snakebite and its consequences. Envenoming from venomous animal bites were mentioned by the WHO [66] as one of the neglected tropical diseases (NTD), due to its frequent occurrence in remote rural areas, and the threat to life because of the absence of the possibility of reaching conventional medical help in the critical period after a bite. Snakebite does not appear among the frequent health problems mentioned for the Ucayali region by the Ministry of Health, because in urban agglomerations that have access to medical treatment, the risk of snakebite is negligible. However, it is often a life-threatening problem in remote rural communities. The plant species used to treat venomous bites and stings are mentioned in detail in the section “Drug activities”.

Pan American Health Organization points out that leishmaniasis is among the top 10 neglected tropical diseases, with more than 12 million people infected globally. Of the nine countries reporting 85% of cases, three are in the Americas: one of them is Peru, where CL is an endemic disease. The available tools for prevention and control are limited, so exposed individuals should take steps to reduce contact with the vector. The Cashinahua people treat incipient and advanced leishmaniasis mainly with preparations from *Jacaranda glabra* (DC.) Bureau & K.Schum. leaves, which, together with *Pityrogramma calomelanos* (L.) Link, *Ruizodendron ovale* (Ruiz & Pav.) R.E.Fr. and *Tanaecium dichotomum* (Jacq.) Kaehler & L. G. Lohmann they also use in the form of fumigation to prevent this disease in the event of an overpopulation of insects, which are its vectors.

That pregnancy, childbirth, and the postpartum period were the most common reasons for hospitalization in the Ucayali region [80] indicates the importance of knowledge about the wide variety of aforementioned plants that Cashinahua women know and use to control these vital processes. Based on informal interviews, we noticed that unless there were major complications, the interviewed women give birth at home, not only because of the distance from the health centre, but mainly because of the different socio-cultural habits and availability of natural resources used to control pregnancy and childbirth.

Our study’s primary contribution is the documentation of indigenous knowledge regarding the treatment of all of the aforementioned health conditions, which are prevalent in Amazonian rural communities. Not only did it help improve public health, especially in remote areas, but it also helped preserve national biocultural heritage, conserve biodiversity, educate allopathic medical professionals about folk medicine, and build community resilience and growth.

## Conclusions

The Cashinahua of the Curanja River live in an area of great botanical diversity that provides effective phytotherapeutic remedies. In the region, people only have limited access to health services and pharmacies. Plants continue to play a significant role due to the remoteness of the region and the lack of other resources. The Cashinahua medical system is affordable, and the nearby forest provides easy access to plant-based remedies. The practice of Cashinahua healers is based on a holistic vision, founded on three fundamental and inseparable pillars: culture, ecology and spirituality. The majority of definitions of ethnobotany focus on uses as a category of interest in ethnobotany. We agree with Monika Kujawska [45] that the method should be understood in a broader sense than just recording and analysing the uses that local communities attribute to plants. We believe that understanding deep and long-lasting relationships between plants and people can be achieved by taking into account ontological categories, nomenclature, plant exchanges and uses.

The exchange of plant material and the knowledge regarding its use has occurred throughout Amazonia. The home gardens of Cashinahua women are used to cultivate aromatic and medicinal herbs, most of which have been brought over from Pucallpa or by their Brazilian kin, for use as spices and as first aid for common childhood ailments. These plants were not presented to us because they do not form part of traditional Cashinahua traditional medicine; however, the fact that they have been introduced and are cultivated and often used directly in these communities is evidence of their proven effects. These introduced plants, grown in small numbers within the community, have never been seen growing wild in the surrounding forest. We suggest that a comprehensive report ought to document the plants that are found in community home gardens.

Another very important area for further research is that of the so-called female plants. Male healers mention gynaecological plants only in the case of disturbed health, i.e. inflammation, neoplasms, excessive or irregular bleeding and haemorrhages. They do not consider plants used by women to treat pregnancy, childbirth and puerperium, because “this is not a disease”. Knowledge of these species is still the domain of Cashinahua women, and an understanding of them and their uses are widespread among them.  We believe that a deeper investigation, including verification of their efficacy or possible toxicity, would be of great benefit.

Previous research has noted an increasing trend in transplanting important or rare medicinal plants from the rainforest to gardens either directly in or near the community, depending on the conditions the plant requires. As we searched in vain among the children of our local collaborators for interest in learning traditional Cashinahua medicine, we noted that the son of one of the main respondents in our research has teamed up with respected *vegetalistas* of his tribe and together they are working to record the uses of their medicinal species in their own language. The ethnobotanical data gathered may serve as the foundation for a policy that promotes community development and the preservation of biodiversity. With careful evaluation and small plantations for the production of medicinal plants for regional use, it is possible to provide inexpensive and effective therapeutic agents as well as an additional source of income to remote areas. We believe that the ethnobotanical data gathered in this study may significantly aid in the preservation of indigenous knowledge about medicinal plants and biodiversity in the area under study.

## Data Availability

The datasets used and/or analysed during the current study are available from the corresponding author on reasonable request.

## Abbreviations

AMETRA: *Proyecto Aplicación de Medicina Tradicional*—Traditional Medicine Application Project
APG IV: The Angiosperm Phylogeny Group IV system of flowering plant classification
CL: Cutaneous leishmaniasis
FECONAPU: *Federación de las Comunidades Nativas de Purús* (Federation of Indigenous Communities of Purus)
IIAP: *Instituto de Investigaciones de la Amazonia Peruana* (Peruvian Amazon Research Institute)
IUCN: Red List of Threatened Species
IVITA: *Instituto Veterinario de Investigaciones Tropicales y de Altura* (Veterinary Institute for Tropical and High Altitude Research)
NTD: Neglected Tropical Diseases
UNIA: *Universidad Nacional Intercultural de la Amazonía* (National Intercultural University of the Amazon)
WoS: Web of Science
